# Attachment Theory: Novel Clinical and Molecular Insights

**DOI:** 10.3390/biom16030452

**Published:** 2026-03-17

**Authors:** Zoë A. MacDowell Kaswan, Lauryn Giuliano, Arie Kaffman

**Affiliations:** Department of Psychiatry, Yale University School of Medicine, 300 George Street, Suite 901, New Haven, CT 06511, USA; zoe.macdowellkaswan@yale.edu (Z.A.M.K.); lauryn.giuliano@yale.edu (L.G.)

**Keywords:** attachment, early-life adversity, animal models

## Abstract

Early-life adversity (ELA) disrupts brain development and is linked to poor health outcomes across species, including humans and rodents. A growing body of work suggests that impaired attachment to a caregiver—arising from erratic, neglectful, or abusive parenting—mediates a substantial portion of ELA’s long-term effects. Despite the conceptual and clinical appeal of this idea, the neural mechanisms by which ELA disrupts attachment and how altered attachment in turn produces diverse psychiatric and medical sequelae remain incompletely understood. In this review, we synthesize recent randomized controlled trials showing that strengthening caregiver–child attachment can ameliorate a broad range of ELA-related outcomes. We also highlight key animal studies that illuminate the biology of attachment and outline critical priorities for future research.

## 1. Introduction

Early-life adversity (ELA) refers to a broad range of negative experiences—including abuse, neglect, chronic poverty, household instability, and exposure to violence—that substantially increase the risk for psychiatric and medical disorders across the lifespan [1,2,3,4,5,6,7]. ELA has been associated with elevated risk for depression, anxiety, psychosis, substance use disorder, cognitive deficits, cardiovascular diseases, arthritis, metabolic syndrome, cancer, and generally reduced life expectancy [8,9,10]. Given that approximately half of all childhood psychopathologies are attributable to ELA [11], there is an urgent need to better understand the underlying biology and to develop more effective interventions [1,2,4,5,6,7].

The primary goal of this review is to illustrate how attachment theory offers a conceptual, clinical, and scientific foundation for elucidating the biological processes through which ELA alters normal development and for informing the development of novel treatments. Attachment theory, first conceptualized by Bowlby [12], posits that reciprocal and predictable interactions between a caregiver and their offspring during a critical period of development establish an evolutionarily conserved emotional bond. This bond promotes appropriate parental investment that is necessary for the survival and normal physiological, cognitive, and emotional development of the offspring [13,14,15,16]. Attachment patterns can be observed and measured using established behavioral paradigms across diverse species. In humans, the most notable method for scoring attachment is Mary Ainsworth’s Strange Situation Procedure (SSP), which evaluates an infant’s responses to separation and reunion with their caregiver and uses systematic behavioral coding to quantify and reliably assess attachment styles [13,17,18,19]. For an excellent illustrative video of the procedure, please see: SSP link, https://www.youtube.com/watch?v=QTsewNrHUHU, accessed on 13 March 2026. Extensions of this framework, along with meta-analytic approaches, now allow researchers to examine attachment patterns during later stages of development past infancy, including preschool and early childhood [19,20].

Many forms of ELA disrupt normative attachment patterns, and early attachment assessments like the SSP have been shown to predict broad psychological, relational, physiological, and cognitive outcomes later in life [19,21]. Importantly, randomized controlled trials (RCTs) that improve attachment security have demonstrated beneficial effects on multiple psychiatric and medical sequelae in high-risk ELA populations [21,22,23,24]. Together, these observations demonstrate the clinical utility of attachment-based interventions and underscore the importance of understanding the biological mechanisms through which ELA disrupts attachment and influences long-term health outcomes.

While human RCTs have yielded critical insights, they cannot directly address the cellular and molecular mechanisms of underlying attachment development or therapeutic response. Animal models offer a powerful complementary approach, as attachment is an evolutionarily conserved phenomenon observed across species, including mammals and some birds [13,15,25]. Studies using rodent, primate, and avian models have provided valuable insights into the neural, hormonal, and behavioral mechanisms of attachment [15,25,26], but relatively few have directly examined how ELA alters attachment development. This represents a critical gap in the field.

Although prior reviews have addressed components of these topics [26,27,28,29], there remains a need to more clearly articulate the clinical relevance of attachment-based interventions and to integrate these insights with mechanistic findings from animal studies. We begin by examining how attachment influences outcomes in the context of ELA, highlighting key clinical lessons from recent attachment-based randomized controlled trials. We then turn to animal studies that provide mechanistic and causal insights into how ELA disrupts attachment to the dam and how these disruptions influence long-term health-related outcomes, along with their clinical implications. We conclude by reviewing emerging insights into the neural circuitry mediating reciprocal caregiver–offspring interactions and by outlining critical future directions for this growing and essential area of research.

## 2. Attachment as a Mediator of ELA-Related Health Outcomes

### 2.1. Function and Subtypes of Attachment

Attachment theory seeks to explain how emotional bonds between infants and their primary caregivers are formed and how these bonds shape psychological and physiological outcomes across the lifespan [12]. The theory integrates insights from ethology, psychoanalysis, and control systems theory and was further informed by William Blatz’s security theory, which emphasized the caregiver’s role as a reliable source of safety that enables confident exploration of the social and physical environment [13,30]. In this framework, caregivers who consistently meet their offspring’s physiological, emotional, and cognitive needs promote the development of internal working models and neural circuits that support a subjective sense of security, trust in others, effective emotion regulation, modulation of stress and immune responses, and healthy cognitive functioning [13,14,15,16].

This conceptual framework was empirically supported by Ainsworth’s observational studies using the SSP [14,17,30]. The SSP is a structured behavioral paradigm that evaluates infant responses to caregiver separation and reunion and was instrumental in identifying four attachment patterns. Secure attachment is characterized by effective caregiver-mediated distress reduction upon reunion and is typically observed when caregivers consistently meet the child’s emotional and physical needs. In contrast, anxiously attached children show heightened distress during separation and difficulty calming upon reunion, whereas avoidantly attached children display minimal distress and may avoid or reject the caregiver during reunion. Children with disorganized attachment demonstrate a combination of anxious and avoidant behaviors [17,18,19]. The latter three patterns are generally classified as insecure attachment, with prevalence rates reaching up to 35% among children exposed to significant neglect or maltreatment [31]. Avoidant attachment is particularly associated with severe neglect [32,33,34,35], potentially reflecting adaptation to repeated caregiver unavailability. Anxious attachment often emerges in contexts of inconsistent caregiving and may also reflect genetic contributions [36]. However, the mechanisms determining why some children develop anxious versus avoidant attachment remain poorly understood. Further work is needed to clarify how genetic vulnerability interacts with distinct forms of adversity to shape attachment subtype [36] and how these patterns relate to circuit-level alterations measurable with resting-state fMRI or diffusion MRI.

### 2.2. Insecure Attachment as a Key Mediator of ELA-Related Outcomes

Secure attachment facilitates the formation of a psychological and emotional “secure base,” enabling children to explore their environment, engage in play and learning, and form healthy social relationships—processes critical for cognitive and emotional development [37]. Secure attachment has also been linked to improved emotional regulation, increased resilience, healthier peer relationships, and better long-term mental and physical health outcomes [13,20,24,38,39]. In contrast, insecure attachment is associated with relationship difficulties and increased rates of attention deficits, depression, anxiety, and substance use later in life [13,20,21,24,28]. Insecure attachment also increases the risk for multiple medical comorbidities, including stunted growth, cardiovascular diseases, diabetes, pain, and a host of chronic inflammatory conditions such as arthritis, ulcerative colitis, and asthma [28,38,39,40].

Importantly, individuals with secure attachment profiles show greater resilience, exhibiting fewer psychiatric symptoms when exposed to comparable levels of adversity relative to insecurely attached peers [41,42,43,44]. Mediation analyses indicate that attachment styles partially explain the association between ELA and adverse health outcomes. Both anxious and avoidant attachment partially mediate the relationship between ELA exposure and adult anxiety, depression, reduced self-esteem, and lower life satisfaction [34,41,44,45].

The strongest evidence that secure attachment mitigates ELA-related risk comes from attachment-based RCTs [21,22,23,24,31,41,42,43,46,47,48]. These studies addressed two critical questions: whether attachment security can be increased in high-risk populations and whether such changes improve health-related outcomes. Both questions have been answered affirmatively. For example, Toth and colleagues demonstrated that weekly toddler–parent psychotherapy significantly improved attachment security in toddlers of depressed mothers compared with untreated controls, with treated children exhibiting even higher attachment scores than those of non-depressed mothers [49]. A follow-up study approximately seven years later showed sustained improvements in attachment security and peer relationships [50].

Additional RCTs indicate that attachment-informed interventions aimed at enhancing caregiver sensitivity reduce psychiatric symptoms and improve executive function, cognitive performance, and overall well-being [21,31,41,42,43,46,47,48]. Notably, attachment-based interventions have also been shown to reduce inflammation [51,52,53,54] and growth stunting [55], suggesting that they confer physiological protection in addition to psychological benefits. For example, Ross and colleagues evaluated the Attachment and Child Health (ATTACH) psychoeducational program, previously shown to improve attachment security in high-risk families [56], and found reduced expression of proinflammatory immune-related genes in both mothers and children exposed to ELA [51].

## 3. Lessons from Animal Studies

### 3.1. Rationale for Using Animal Models

Randomized controlled trials provide compelling evidence that attachment-based interventions strengthen caregiver–child relationships and confer lasting mental and physical health benefits following ELA [21,22,23,24,31,41,42,43,46,47,48]. Although the cellular and molecular mechanisms linking attachment to such broad outcomes are difficult to study directly in humans, these questions are well suited for investigation in animal models because attachment is a highly conserved phenomenon across different cultures [57,58] and mammalian species, including humans and rodents [12,26,29]. This foundational principle, articulated by Bowlby [12], has been reinforced by observational paradigms, such as the SSP allowing for objective quantification of attachment-related behaviors—such as proximity seeking and distress vocalizations following separation from a caregiver or dam—across species [12,26,29]. Importantly, the ability to operationalize and quantify attachment behavior using objective behavioral metrics distinguishes this construct from many psychiatric diagnoses, which rely heavily on subjective symptom reporting and culturally influenced criteria [7].

Rodent models allow precise control over key variables that are challenging to manipulate in humans, such as the timing and severity of ELA, genetic background, and access to brain tissue during sensitive developmental periods. Moreover, experimental strategies that enable monitoring and manipulation of defined cell populations—including calcium imaging, optogenetics, chemogenetics, and genetic gain- or loss-of-function approaches—permit causal tests linking ELA-induced alterations in maternal care to deficits in attachment-related behaviors and long-term health outcomes [7,59]. This is especially important given recent technical advances in our ability to record and manipulate neuronal activity from awake behaving rodent pups [60,61,62,63,64] and non-invasive imaging studies such as rsfMRI or dMRI that can be used across species [7,65].

Despite strong evidence supporting a central role for attachment in shaping lifelong health, many fundamental questions remain unanswered. For example, we have a limited understanding of how distinct forms of ELA disrupt attachment development, how alterations in attachment-related circuitry influence defensive behavior, reward processing, immune function, and cognitive flexibility later in life, or how subsequent life experiences further modify these trajectories. In the following sections, we highlight key mechanistic studies that begin to address these gaps, emphasizing unifying concepts and their relevance to clinical observations in humans.

### 3.2. The Magic of Touch

**Figure 1:**
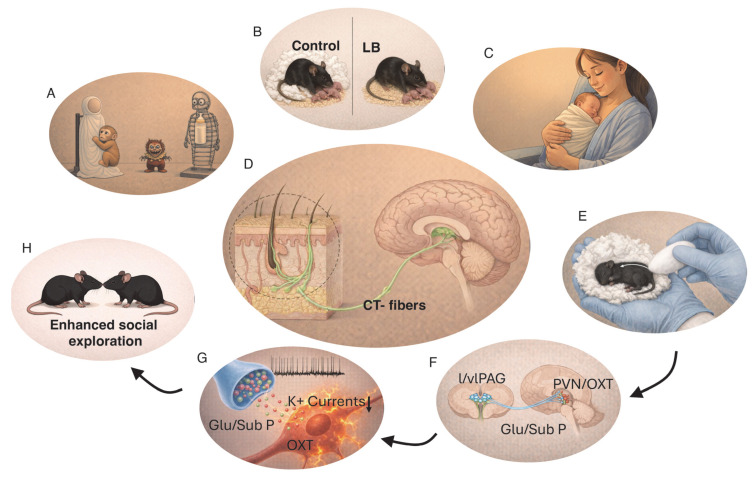
Early-life activation of CT fibers induces sustained oxytocin release and enhanced social exploration later in life. (**A**) Schematic representation of Harlow’s experiments showing that infant macaques preferentially cling to a cloth surrogate rather than a wire surrogate when confronted with a novel threat. (**B**) Rodent pups raised under limited bedding (LB) conditions, characterized by the absence of nesting material (white fluffy material), display avoidant-like attachment behavior toward an anesthetized dam. (**C**) Skin-to-skin contact provided by kangaroo care enhances attachment and improves a broad range of health-related outcomes. (**D**) Comfort touch is mediated by C-tactile (CT) fibers. (**E**–**H**) Stroking mouse pups at velocities that optimally activate CT fibers (**E**) induces neuronal activation in the lateral/ventrolateral periaqueductal gray (l/vlPAG). Activation of l/vlPAG neurons triggers the release of glutamate and substance P onto oxytocin-producing neurons in the paraventricular nucleus (PVN), resulting in reduced transient K^+^ currents and increased spontaneous firing of oxytocin-positive neurons (**F**,**G**). These electrophysiological changes persist into later life and are associated with enhanced social exploration (**H**). CTL = control, OXT = oxytocin, Glu/Sub = Glutamate and Substance P. down arrow = reduction.

Comfort touch is believed to play a particularly important role in the development of maternal attachment [9,66]. As with many foundational scientific insights, this idea emerged from a striking and initially puzzling observation. In the 1950s, Harlow and colleagues noted that infant macaques raised without their mothers formed strong attachments to a soft cheesecloth blanket placed over the wire floor of their cages and became markedly distressed when the blanket was removed [67]. This observation led to a series of classic experiments demonstrating the primacy of comfort over nourishment in establishing an attachment bond capable of regulating infant distress (Figure 1A). When given access to both a cloth-covered surrogate mother and a wire surrogate mother that provided food, infant macaques spent most of their time with the cloth surrogate and were only able to regulate their responses to fearful stimuli in its presence [67]. In contrast, infants raised exclusively with a wire surrogate displayed pronounced emotional dysregulation when exposed to novel fearful objects and developed avoidant attachment toward the wire mother [67]. Notably, avoidant attachment–like behaviors have also been observed in rodents raised in the absence of soft nesting material [59,64] (Figure 1B), suggesting that the role of comfort touch in supporting secure attachment reflects an evolutionarily conserved mechanism.

The relationship between comforting touch and fearfulness has been further examined using mouse and rat models. Natural variation in maternal care can be quantified by measuring dams’ levels of licking and grooming (LG) and arched-back nursing (ABN) during the first postnatal week, allowing classification of dams as providing either high or low levels of care (high-LG/ABN vs. low-LG/ABN). Offspring of high-LG/ABN dams exhibit reduced fearfulness and attenuated hypothalamic–pituitary–adrenal (HPA) axis reactivity compared with offspring of low-LG/ABN dams [68,69,70]. Importantly, these effects are fully reversed by cross-fostering, demonstrating a causal role for maternal care rather than genetic inheritance [69]. Experimental manipulation of tactile stimulation further supports this conclusion. Mimicking maternal LG by gently stroking pups with a paintbrush—but not by other forms of tactile stimulation—recapitulates the effects of high maternal care on stress reactivity and reverses the consequences of maternal deprivation [71,72,73,74]. Subsequent work demonstrated that levels of LG during the first postnatal week program long-term HPA axis function by inducing stable epigenetic modifications, including changes in DNA methylation and expression of stress-regulatory genes such as the glucocorticoid receptor [9,75].

These studies delineated a central role for comfort touch in the development of core features of secure attachment, including proximity-seeking behavior, use of the caregiver as a secure base for exploration, and the capacity to regulate emotional distress [13,14,15,16]. In humans, skin-to-skin contact between mother and infant (“kangaroo care”, Figure 1C) has been shown to reduce physiological stress markers in infants in neonatal intensive care units [76] and to enhance attachment scores [77]. A meta-analysis of 124 studies further demonstrated that skin-to-skin contact reduces the risk of sepsis and mortality and improves multiple health-related outcomes and growth [78], as well as executive function later in life [77].

A growing body of work suggests that activation of peripheral C-tactile (CT) fibers—also referred to as low-threshold mechanosensitive C fibers—plays a critical role in mediating the effects of comfort touch on the development of secure attachment, reviewed in [66,79,80]. These evolutionarily conserved mechanoreceptors are found in hairy skin and respond selectively to gentle stroking (Figure 1D). In humans, CT fibers are optimally activated by low-force stroking at approximately 3 cm/s and at a temperature of ~32 °C, corresponding to the surface temperature of a human hand. CT fibers are functional early in development, and their activation promotes parasympathetic tone, leading to reductions in heart rate, cortisol levels, and pain—effects that closely parallel those observed following skin-to-skin contact [66,79,80]. Importantly, stimulation of CT fibers is experienced as rewarding, activates brain regions involved in social perception [79], and triggers the release of oxytocin [81,82]. In contrast, CT-fiber stimulation in individuals with disorganized attachment is perceived as less pleasant and is associated with aberrant activation of threat-related limbic regions, including the amygdala [83]. Abnormal sensory responses to CT-fiber activation are also widely observed in individuals with severe autism [66], further supporting a role for these fibers in promoting secure attachment.

Elegant studies in mice have established a causal link between early-life CT-fiber activation, oxytocin release, and long-term changes in social affiliation and the rewarding effects of oxytocin [81]. In these experiments, 15-day-old mouse pups were placed in a cotton nest held in the palm of a researcher’s hand and gently stroked with a cotton ball at frequencies that selectively activate CT fibers, four times daily for 3–5 days (Figure 1E). This procedure increased oxytocin expression in the paraventricular nucleus (PVN) of the hypothalamus and elevated the spontaneous firing rate of oxytocin-positive neurons (Figure 1F,G). Remarkably, these electrophysiological changes persisted into adulthood and were associated with increased social exploration (Figure 1H) and a conditioned place preference for cotton bedding over home-cage bedding, indicating that the tactile stimulation itself acquired rewarding properties. These effects were not observed in pups exposed to identical handling without stroking or when stroking was delivered at higher, non-CT-activating frequencies, demonstrating the specificity of the response to CT-fiber activation [81]. Critically, stroking failed to enhance social affiliation or cotton preference in oxytocin-knockout mice, while daily chemogenetic activation of PVN oxytocin neurons from postnatal day 15 to 20 was sufficient to recapitulate the behavioral and physiological effects of stroking [81]. Together, these findings establish that oxytocin release is necessary for the long-term behavioral consequences of CT-fiber activation and that early-life activation of oxytocin-positive neurons is sufficient to induce these enduring changes. Mechanistically, the sustained increase in spontaneous firing of oxytocin neurons was attributed to a reduction in transient K^+^ currents, driven by synaptic release of glutamate and substance P from afferent neurons located in the lateral and ventrolateral periaqueductal gray (l/vlPAG) [81] (Figure 1G).

Given that individuals with disorganized attachment exhibit altered responses to CT-fiber stimulation [83], an important open question is whether similar stroking paradigms would elicit comparable outcomes in pups displaying disorganized-like attachment and whether such interventions could enhance secure-like attachment to the dam in models of ELA (see Section 3.4 below). Finally, the cellular and molecular mechanisms underlying the long-term reduction in transient K^+^ currents in oxytocin neurons remain to be elucidated. In particular, it will be important to determine whether these changes are mediated by stable chromatin and gene-expression modifications, analogous to those described by Michael Meaney’s group in response to natural variation in maternal LB/ABN behavior, reviewed in [9,75].

### 3.3. Mechanistic Lessons from Rats Highlight a Critical Role for Elevated Corticosterone and Aberrant Amygdala Activation

Exposure to limited bedding (LB) during early postnatal development leads to erratic and occasionally abusive maternal behaviors [59,84], including dragging or stepping on pups [63,64]. Studies using this rodent model have provided some of the most compelling mechanistic insights into how ELA disrupts attachment-like behaviors, which are briefly summarized here.

**Figure 2:**
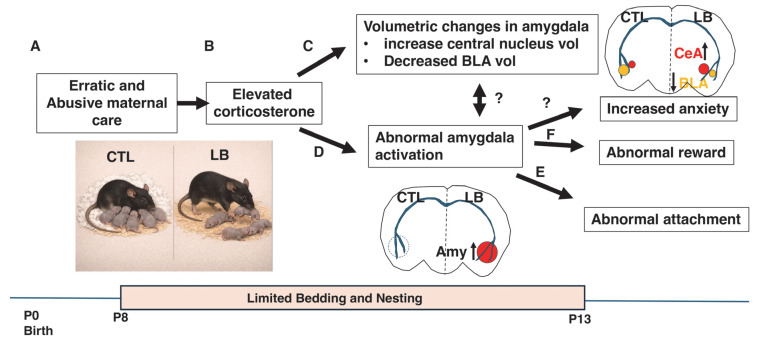
Elevated corticosterone induced by maternal maltreatment disrupts amygdala activation in response to maternal cues and promotes avoidant attachment. Exposure to limited bedding (LB) conditions induces erratic maternal care (**A**) and elevates corticosterone levels (**B**) during the stress hyporesponsive period, a developmental window in which corticosterone levels are normally low. Elevated corticosterone leads to volumetric alterations in the developing amygdala (**C**) and aberrant amygdala activation in response to an anesthetized dam in LB pups (**D**); right hemisphere, effects that are not observed in control (CTL) pups (left hemisphere). This abnormal amygdala activation underlies avoidant attachment behavior at postnatal day 13 (**E**) and contributes to long-term deficits in reward-related behaviors later in life (**F**). CTL = control, Amy = amygdala, up-pointing arrow = increase, Down-pointing arrow = decrease.

Raineki et al. (2019) showed that LB exposure from postnatal day (P)8–P13 induces fragmented and abusive maternal care that is associated with elevated corticosterone levels in pups [64] (Figure 2A,B). At P13, LB pups displayed abnormal proximity-seeking behavior toward an anesthetized dam, characterized by increased time spent on the dorsal rather than the ventral surface of the dam and reduced nipple attachment. This atypical approach behavior was accompanied by heightened amygdala activation in the presence of the dam and structural remodeling of the amygdala, including a reduced basolateral nucleus and an expanded central nucleus [64] (Figure 2C,D). Together, these findings suggested that maternal cues had acquired an aversive valence in LB pups, resulting in avoidant-like attachment behavior (Figure 2E).

Pharmacological manipulations demonstrated a causal role for corticosterone in these effects. Blocking corticosterone signaling normalized approach behavior in LB pups, whereas daily corticosterone administration to control pups—in the presence of an awake or anesthetized dam, but not a nonsocial stimulus—recapitulated the LB phenotype. This included abnormal maternal approach behavior, volumetric changes in the amygdala, and heightened amygdala activation. Importantly, direct inhibition of amygdala activity via muscimol infusion prior to testing restored normal maternal approach behavior in pups receiving daily corticosterone injections [64]. Collectively, these findings indicate that maltreatment leads to repeated pairing of elevated corticosterone with maternal cues early in life, driving amygdala remodeling and aberrant amygdala activation in response to maternal stimuli. This maladaptive neural response underlies the avoidant-like attachment deficits observed in LB pups (Figure 2).

In addition to altering responses to maternal cues, LB attenuated cortical responses to nurturing behaviors such as licking and grooming and arched-back nursing—deficits that were reversed by blocking corticosterone [63]. These findings are particularly notable given that the first ~12 days of life constitute the stress hyporesponsive period, during which basal corticosterone levels are low and relatively unresponsive to stress, especially in the presence of maternal cues [85,86]. A critical function of maternal care and secure attachment during this period is therefore to buffer pups against corticosterone elevations. Repeated failures of this buffering mechanism appear to erode the rewarding valence of nurturing maternal cues and promote aversive responses even to passive maternal stimuli, such as those emitted by an anesthetized dam (Figure 2).

Complementary work from the Baram laboratory has shown that abnormal amygdala responses extend beyond maternal cues to other naturally rewarding stimuli later in life [87] (Figure 2F). Young adult male rats exposed to LB exhibited reduced social play and decreased sucrose consumption, indicative of anhedonia, which persisted into adulthood. These behavioral deficits were accompanied by aberrant activation of corticotropin-releasing hormone (CRH)–positive neurons in the central amygdala. Viral knockdown of CRH expression in the amygdala reversed LB-induced anhedonia, establishing a causal link between early adversity, amygdala CRH signaling, and long-term reward deficits [87]. These findings align closely with human neuroimaging studies demonstrating altered amygdala activation and disrupted reward processing in individuals exposed to ELA [88,89].

### 3.4. Beyond Corticosterone: Insights from Mice

Most studies examining the effects of LB on maternal attachment have been conducted in rats. While highly informative, rat models are constrained by a relative lack of genetic tools, limiting the ability to dissect molecular mechanisms and cell-type-specific contributions to attachment-related behaviors [59]. Extending this work to mice enables the use of powerful genetic, genomic, and molecular tools that are not readily available in rats and facilitates integration with recent mouse studies that have identified key mechanisms governing adult social behavior as well as how pups normally perceive and respond to maternal cues (see below).

In addition, there is a critical need for more rigorous characterization of maternal care in the LB paradigm using continuous, 24/7 home-cage monitoring, as well as for improved behavioral assays to assess maternal attachment across developmental stages [59]. To address these gaps, we used Phenotyper cages to continuously monitor pup–dam interactions in control and LB mouse litters during the first postnatal week. Although previous studies have employed video monitoring to assess maternal behavior in LB mice, these analyses were typically limited to brief observation windows rather than continuous recording, and none examined how erratic maternal behavior impacts attachment-like behavior in pups [59].

Using this approach, we confirmed that LB induces highly fragmented maternal care that was more pronounced during the dark cycle and characterized by frequent nest entries and exits. We also reported for the first time that LB dams displayed elevated levels of stretch-attend defensive postures while on the nest, as well as significantly increased locomotor activity. These abnormal maternal behaviors were strongly correlated with growth retardation that persisted into adolescence and was more pronounced in males [59]. These findings parallel human studies demonstrating growth stunting in severe forms of childhood neglect and showing that attachment-based interventions can enhance growth and reduce stunting [55].

**Figure 3:**
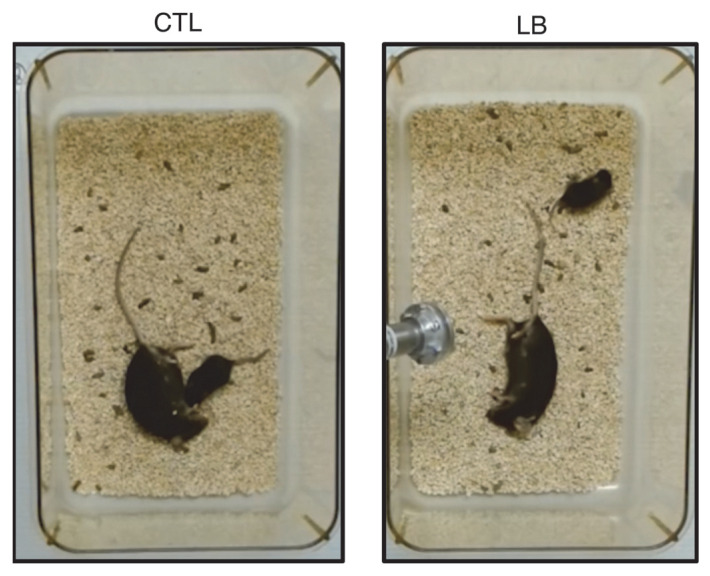
Thirteen-day-old control (CTL) and limited bedding (LB) pups were placed with their anesthetized dam for 5 min to assess proximity-seeking behavior and record ultrasonic vocalizations (USVs; analogous to distress calls) using a specialized microphone (shown in the LB image). Dams were anesthetized to observe pup-directed behavior and vocalizations. CTL pups exhibited the expected approach behavior toward the dam, whereas LB pups did not. Additional methodological details, quantification, and videos of this behavior are available in our previous work [59].

Consistent with findings in rats, LB elevated corticosterone levels in mouse pups and was associated with abnormal approach behavior toward an anesthetized dam at P13 (Figure 3). Notably, only approximately half of LB pups within a given litter exhibited deficits in maternal approach, revealing unexpected individual variability in attachment-like behavior that warrants further investigation [59]. Maternal-directed behaviors were also assessed at P8 and P18. At P8, LB pups emitted fewer ultrasonic vocalizations (USVs) during a 5 min isolation period compared with controls; however, maternal buffering—defined as the reduction in vocalizations upon contact with a maternal figure—was preserved. At P18, LB pups displayed increased anxiety-like behavior in the open field, a finding replicated across multiple cohorts. Despite this heightened anxiety-like behavior, LB pups retained a robust preference for their dam over a novel object (“maternal preference”) [59]. To our knowledge, this represents the first demonstration that LB induces avoidant-like attachment deficits in mice. Importantly, despite these abnormalities and elevated pup corticosterone, maternal buffering at P8 and maternal preference at P18 remained intact, underscoring the resilience of this fundamental bond [59].

**Figure 4:**
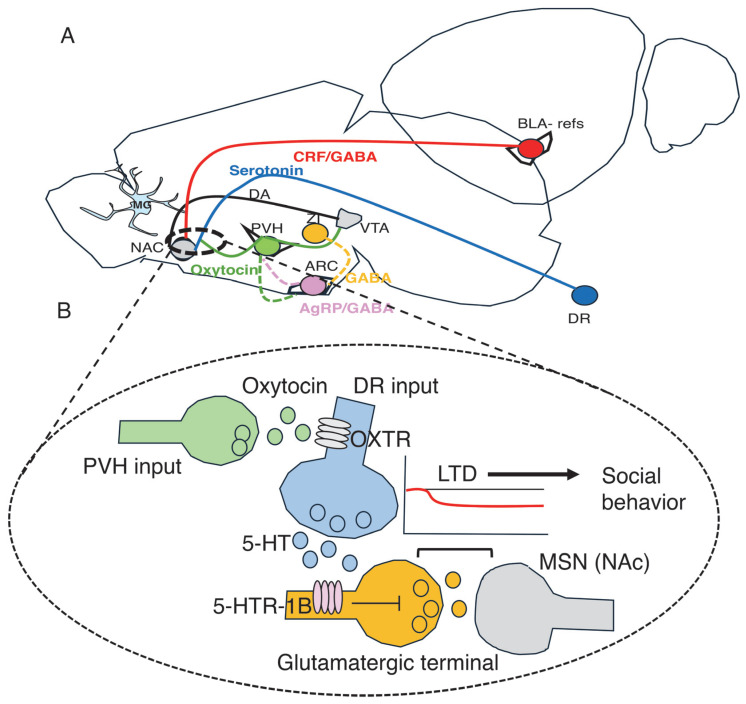
Mouse studies identify key brain regions and circuits that enable pups to detect and respond to maternal cues and support social behavior later in life. (**A**) Somatostatin-positive neurons in the zona incerta (ZI, yellow) are selectively activated by maternal cues, and their activation recapitulates outcomes observed following reunion with the dam, including reduced USVs and decreased corticosterone levels [61]. Maternal separation activates AgRP (purple)- and oxytocin-positive neurons (green). Activation of AgRP neurons increases USVs (distress-related vocalizations) [62], whereas activation of oxytocin neurons promotes social proximity and alters USV patterns upon reunion with the dam [60]. Activation of AgRP [90] and oxytocin neurons [92] also contributes to social behavior during adolescence but plays a lesser role in fully adult mice. Oxytocin neurons coordinate social behavior by directly modulating serotonin-mediated transmission in the nucleus accumbens (NAc) [91,92] and indirectly by enhancing dopamine release in the NAc via projections to the ventral tegmental area (VTA) [93]. In addition, GABAergic innervation from corticotropin-releasing factor (CRF)-expressing neurons (red) in the basolateral amygdala (BLA) alters social and reward sensitivity later in life, particularly in males [87,100]. Exposure to limited bedding (LB) impairs microglia-mediated synaptic pruning during a critical developmental window, resulting in abnormal circuit connectivity and behavioral outcomes later in life [101]. These microglial deficits may also contribute to disrupted connectivity within circuits supporting secure attachment. DA = dopamine. (**B**) Serotonergic projections from the dorsal raphe (DR; blue axon terminals, blue line in A) activate 5-HT1B receptors (purple) located on glutamatergic terminals (yellow) that innervate medium spiny neurons (MSNs) in the nucleus accumbens (gray dendritic spine). Activation of 5-HT1B receptors suppresses glutamate release, inducing LTD at MSN synapses that regulate social behavior during adolescence but not adulthood [98]. Oxytocin-positive terminals originating from the paraventricular nucleus of the hypothalamus (PVH; green) modulate serotonin release via oxytocin receptors expressed on serotonergic terminals [91,92].

The difficulty of fully eroding attachment behavior is consistent with its essential role in ensuring pup survival and suggests the presence of redundant and developmentally regulated circuitry. Recent studies in mice have provided important new insights into the complexity with which normally developing pups process and respond to maternal cues, opening new avenues to test whether maladaptive development of these circuits underlies attachment deficits in LB pups (Figure 4A).

For example, somatostatin-expressing neurons in the zona incerta (ZI)—a subthalamic region involved in sensory integration—are selectively activated by interactions with the dam in P16–18 pups, suggesting that they encode a neural representation of the mother (Figure 4A). Activation of these neurons suppresses isolation-induced USVs in P11 pups and attenuates corticosterone responses at P15 [61]. It will be important to determine whether LB reduces the ability of maternal cues to activate these maternally responsive somatostatin neurons in the ZI.

The arcuate nucleus (ArcN), traditionally studied for its role in adult energy balance, has also emerged as a key node in processing maternal cues during early development. Agouti-related peptide (AgRP)-expressing neurons in the ArcN are activated by maternal separation and promote USV emission (Figure 4A). Calcium imaging studies demonstrate that these neurons are rapidly inhibited by reunion with the dam or by warmth—but not by milk—indicating a role in social rather than nutritional signaling [62]. Chemogenetic activation of AgRP neurons is sufficient to induce USVs and enhance maternal attention, establishing a causal role in proximity-seeking behavior [62]. Moreover, prolonged social isolation enhances social interaction upon reunion in juvenile—but not adult—mice, a process that requires AgRP neuron activation [90]. Together, these findings highlight a prominent role for AgRP neurons in proximity-seeking behaviors during early postnatal life and adolescence. LB reduces USVs during maternal separation and exploration of an anesthetized dam, consistent with an avoidant-like attachment phenotype [59]. It will therefore be important to test whether LB blunts AgRP activation and rebound socialization following isolation during adolescence.

As discussed above, comfort touch in P15 pups induces long-lasting increases in oxytocin neuron activity and enhances social motivation later in life [81] (Figure 1). Recent calcium imaging studies have shown that maternal separation produces sustained activation of oxytocin-expressing neurons in P15 pups, which is necessary for proximity-seeking behavior upon reunion with the dam [60]. Activation of oxytocin neurons precedes USV emission, suggesting that these neurons act downstream of AgRP-positive cells [60] (Figure 4A). Later in life, oxytocin neurons in the paraventricular nucleus (PVN) are selectively activated by social cues, and this activation is both necessary and sufficient to support the rewarding aspects of social affiliation [91,92]. Oxytocin orchestrates social reward by facilitating serotonergic-mediated long-term depression (LTD) in medium spiny neurons (MSNs) of the nucleus accumbens (NAc, Figure 4B) [91,92] and by activating dopaminergic neurons in the ventral tegmental area (VTA) [93], leading to dopamine release in the NAc (Figure 4A).

Human studies have reported hypermethylation of the oxytocin gene or its receptor in association with ELA [94] and insecure attachment [95,96,97]. However, these studies relied on peripheral tissues, and it remains unclear whether such epigenetic signatures reflect changes within the relevant neuronal populations in the brain. projections from the dorsal raphe (DR; blue axon terminals) activate 5-HT1B receptors (purple) located on glutamatergic terminals (yellow) that innervate medium spiny neurons (MSNs) in the nucleus accumbens (gray dendritic spine). Activation of 5-HT1B receptors suppresses glutamate release, inducing LTD at MSN synapses that regulate social behavior during adolescence but not adulthood [98]. Oxytocin-positive terminals originating from the paraventricular nucleus of the hypothalamus (PVH; green) modulate serotonin release via oxytocin receptors expressed on serotonergic terminals [91,92].

Oxytocin-dependent enhancement of social reward and electrophysiological changes in MSNs (Figure 4B) were observed in adolescent but not adult mice [92], mirroring the developmental specificity of AgRP-mediated social rebound following isolation [90]. These findings point to a developmental shift in the influence of these circuits on affiliative behavior, which may help explain how ELA disrupts maternal attachment and social reward during adolescence while still allowing for plasticity in adult attachment [99].

Finally, serotonergic neurons in the dorsal raphe nucleus are highly sensitive to social cues and bidirectionally regulate glutamatergic inputs onto NAc MSNs via the 5-HT1B receptor [98] (Figure 4B). Manipulations that enhance serotonergic signaling—such as optogenetic activation or administration of 5-HT1B receptor agonists in the NAc—promote social exploration, whereas optogenetic inhibition or 5-HT1B receptor blockade reduces social behavior (Figure 4B). Notably, serotonergic modulation of the NAc is selectively rewarding for social stimuli and, unlike dopaminergic signaling, does not increase operant responding or intracranial self-stimulation [98]. Whether these serotonergic pathways contribute to maternal attachment or are altered by LB remains an important open question.

## 4. Future Directions

Recent advances in our understanding of the neural circuitry underlying social reward in adulthood and the processing of maternal cues during early development, together with the ability to rigorously quantify pup–dam interactions using automated home-cage monitoring, open exciting new opportunities to dissect how ELA disrupts maternal attachment. These approaches now allow abnormal maternal behaviors to be directly linked to attachment-like deficits, providing a powerful framework to identify underlying mechanisms.

A key priority is to determine how erratic maternal care in the LB model alters neuronal activity and genomic regulation within circuits that encode maternal cues. In particular, future studies should examine whether LB affects the function and transcriptional landscape of somatostatin-expressing neurons in the zona incerta [61] and AgRP-expressing neurons in the arcuate nucleus [62], and whether dysregulation of these populations contributes to avoidant-like attachment phenotypes [59]. Parallel investigations should assess oxytocin signaling and serotonergic innervation of the NAc [91,98]. In this context, it will be especially informative to test whether augmenting comfort touch—using gentle stroking [81]—can normalize oxytocin neuron activity and rescue attachment-related behaviors in LB pups.

Recent work from our laboratory and others has demonstrated that LB disrupts microglia-mediated synaptic pruning during a critical developmental window, leading to enduring alterations in synaptic connectivity, cognition, and stress responsivity [101,102,103]. An important next step is to test whether impaired microglial pruning also contributes to aberrant circuit connectivity underlying attachment deficits (Figure 4A). In adolescent LB mice, microglia-associated connectivity abnormalities have been identified using DiOlistic spine labeling, high-resolution confocal imaging of glutamatergic synapses, and resting-state fMRI [101,103]. Unbiased whole-brain voxel-based analyses further revealed reductions in local functional connectivity within regions implicated in social reward, including the NAc, hypothalamus, and the basolateral nucleus of the amygdala [101]. Because findings in rodents do not always directly translate to humans, a major strength of this imaging framework is that analogous neuroimaging approaches can be applied in humans to relate attachment phenotypes to alterations in functional connectivity.

The tightly controlled nature of rodent experiments provides critical insight into the influence of specific variables; however, it also introduces limitations for translatability. Humans are raised in environments characterized by multiple interacting stressors, more complex caregiver relationships (multiple parents, non-parental caregivers), and the capacity to modify caregiving dynamics through intentional behavioral modifications. Furthermore, behavioral analysis–especially of new, less mechanistically validated behavioral paradigms–carry an inherent risk of anthropomorphism. Nevertheless, the current data strongly suggest that the LB rodent model captures key behavioral aspects of attachment-related deficits, while acknowledging the constraints inherent to cross-species extrapolation.

Additional work is needed to clarify how specific aberrations in maternal care differentially impact attachment behavior. For example, overtly abusive behaviors—such as stepping on, kicking, or dragging pups—may lead to outcomes distinct from those resulting from erratic or inconsistent maternal availability. Our ability to continuously monitor maternal behavior using a 24/7 automated video system will provide important insights into these distinctions.

Further studies are also needed to characterize maternal attachment across multiple developmental stages and to identify mechanisms underlying stable individual differences in attachment outcomes. These efforts should include assessing long-term epigenetic modifications within relevant neuronal populations and determining whether parallel signatures can be detected in accessible peripheral tissues, such as blood or buccal epithelial cells. Finally, it will be essential to evaluate the translational relevance of mechanistic insights derived from mouse models by extending these studies to nonhuman primate models of ELA.

## 5. Concluding Remarks

Recent randomized controlled trials summarized here underscore the clinical utility of attachment-based interventions in mitigating the medical and psychiatric consequences of ELA. Despite their promise, many fundamental questions remain regarding the mechanisms by which ELA disrupts maternal attachment and how these disruptions contribute to adverse health outcomes later in life. Ongoing advances in mouse models are beginning to clarify these mechanisms and will provide a critical foundation for translational studies in nonhuman primates and, ultimately, in humans.

## Data Availability

No new data were created or analyzed in this study. Data sharing is not applicable to this article.

## Abbreviations

The following abbreviations are used in this manuscript:

ABN	Arched back nursing
AgRP	Agouti-related peptide
ArcN	Arcuate nucleus of the hypothalamus
ELA	Early-life adversity
CT	c-tactile fibers
CTL	Control
LB	Limited bedding
HPA axis	hypothalamic–pituitary–adrenal axis
LG	Licking and grooming
LTD	Long-term depression
MSNs	Medium spiny neurons
NAc	Nucleus accumbens
P	Postnatal day
PVN	Paraventricular nucleus of the hypothalamus
RCTs	Randomized controlled trials
SSP	Strange Situation Procedure
USVs	Ultrasonic vocalizations
VTA	Ventral tegmental area
ZI	Zona incerta
